# Summated Hazard Score as a Powerful Predictor of Fatigue in Relation to Pacing Strategy

**DOI:** 10.3390/ijerph18041984

**Published:** 2021-02-18

**Authors:** Sylvia Binkley, Carl Foster, Cristina Cortis, Jos J. de Koning, Christopher Dodge, Scott T. Doberstein, Andrea Fusco, Salvador J. Jaime, John P. Porcari

**Affiliations:** 1 Department of Exercise and Sport Science, University of Wisconsin-La Crosse, La Crosse, WI 54601, USA; sylbink@gmail.com (S.B.); cdodge@uwlax.edu (C.D.); sdoberstein@uwlax.edu (S.T.D.); sjaime@uwlax.edu (S.J.J.); jporcari@uwlax.edu (J.P.P.); 2 Department of Human Sciences, Society and Health, University of Cassino and Lazio Meridionale, 03043 Cassino, Italy; c.cortis@unicas.it (C.C.); andrea.fusco@unicas.it (A.F.); 3 Department of Human Movement Science, Movement Sciences Amsterdam, Vrije Universiteit, 1081BT Amsterdam, The Netherlands; j.j.de.koning@vu.nl

**Keywords:** pacing, cycling, time trial, RPE, performance

## Abstract

During competitive events, the pacing strategy depends upon how an athlete feels at a specific moment and the distance remaining. It may be expressed as the Hazard Score (HS) with momentary HS being shown to provide a measure of the likelihood of changing power output (PO) within an event and summated HS as a marker of how difficult an event is likely to be perceived to be. This study aimed to manipulate time trial (TT) starting strategies to establish whether the summated HS, as opposed to momentary HS, will improve understanding of performance during a simulated cycling competition. Seven subjects (peak PO: 286 ± 49.7 W) performed two practice 10-km cycling TTs followed by three 10-km TTs with imposed PO (±5% of mean PO achieved during second practice TT and a self-paced TT). PO, rating of perceived exertion (RPE), lactate, heart rate (HR), HS, summated HS, session RPE (sRPE) were collected. Finishing time and mean PO for self-paced (time: 17.51 ± 1.41 min; PO: 234 ± 62.6 W), fast-start (time: 17.72 ± 1.87 min; PO: 230 ± 62.0 W), and slow-start (time: 17.77 ± 1.74 min; PO: 230 ± 62.7) TT were not different. There was a significant interaction between each secondary outcome variable (PO, RPE, lactate, HR, HS, and summated HS) for starting strategy and distance. The evolution of HS reflected the imposed starting strategy, with a reduction in PO following a fast-start, an increased PO following a slow-start with similar HS during the last part of all TTs. The summated HS was strongly correlated with the sRPE of the TTs (r = 0.88). The summated HS was higher with a fast start, indicating greater effort, with limited time advantage. Thus, the HS appears to regulate both PO within a TT, but also the overall impression of the difficulty of a TT.

## 1. Introduction

Pacing is most simply defined as the distribution of energy expenditure over time intended to accomplish a desired goal without excessive fatigue or negative health effects [1,2]. A variety of evidence suggests that appropriate pacing contributes to optimizing performance in time-based athletic events [3,4,5,6]. In head-to-head competition, less successful athletes often follow the pacing pattern of the eventual winner, until they are compelled to change to more individually realistic pacing patterns [7,8]. In events where athletes, either elite or recreational, are improving their own best performances, the same pacing pattern is often adopted [9]. In non-athletic individuals, health complications are associated with unaccustomed heavy exercise [10]. Additionally, training sessions that start out too hard, are often associated with poor adherence in persons training for health and fitness [11]. Therefore, for optimizing performance, preventing health complications and improving adherence with training, proper pacing of exercise bouts is critical.

The basis of pacing reaches back to a hunter–gatherer society, where hunters had to make effort/reward decisions when pursuing game [12]. This problem was shared by migrant groups and armies, with the challenge of achieving goals while avoiding exhaustion. For example, Roman legionnaires were trained to march over twenty miles in a “full step”, while carrying up to 27 kg (~50% body weight) [13]. Since inability to sustain the march pace was punishable by death, managing energy expenditure was critical. Even athletes, performing very challenging tasks such as the grand tours in cycling [14] and systematic training for competition, distribute the relative effort during training such that only 10–20% of training is performed at high intensities [15,16,17]. Similarly, a normal practice for older industrial workers is to “pace” tasks in order to make the workload acceptable [18].

The concept of pacing highlights the importance of controlling intensity throughout an exercise bout in order to avoid unacceptably large homeostatic disturbances [1,2,3,4,5,6,19,20,21]. Further, pacing in athletics may represent the difference between a first-place win and an early-race burnout or between a pleasant [22] exercise session and one that is likely to be perceived as too difficult and is unlikely to be repeated [23]. Robinson et al. [24] performed the first controlled studies of pacing in relation to exercise performance as early as 1958, although there was not widespread interest in pacing until the 1990s. They studied homeostatic disturbances during differently paced middle-distance races with the intent of understanding optimal pacing. This early study laid the groundwork for future pacing strategies, suggesting that for middle distance events it is important to follow a relatively even pace and conclude the event with an “end spurt” in order to optimally utilize energetic reserves [1,2,3].

Contemporary studies have extended this concept by looking at changes in energy expenditure relative to the details of specific athletic competitions. During shorter events, particularly when the primary retarding factor is air resistance (cycling/skating), it appears best to utilize anaerobic energy quickly to compensate for the short race duration, as velocity at the end of a race can be viewed as wasted kinetic energy [25]. The opposite appears to be true in middle and longer distance events, particularly where gravity or water provide the retarding factor (running/swimming), where it is possible to have a large slowdown that negatively impacts performance [4]. Similar results were found by Tucker et al. [26] when analyzing world record performances in 800 m, 5000 m, and 10,000 m running. In the 800 m, greater running speeds were reached during the first lap with a typical slowdown in the second lap. In the 5000 m and 10,000 m, an end spurt was possible because of the maintenance of energy reserves during the middle portion of the race. Similar results were noted in the 2008 Beijing Olympic track races [7]. Noakes et al. [27] noted that in 1-mile running world records, there was a distinct pacing pattern of starting fast, slowing through the middle of the race, and then running faster during the last lap. However, Foster et al. [28] noted that 1-mile running world records had evolved to become much more “even paced” during the last 25 years. Further, Foster et al. noted that when an individual athlete, whether elite or recreational level, bettered their own best performance, they typically used the same relative pacing pattern [9]. Abbiss and Laursen [1] noted the importance of an all-out strategy in shorter races, a positive or gradual decrease in pace after reaching maximum velocity in middle distance events, and an even pacing strategy in longer distance events. Similar evidence was presented by Foster et al. [29] showing that events of different durations had unique pacing profiles. Joseph et al. [30] and Faulkner et al. [31] showed that when time trials (TTs) were normalized to relative distance, all events had a similar structure. This is further supported by the observation that depletion of anaerobically attributable energetic reserves, represented by W’, is responsible for failures to maintain power output (PO) during fatiguing tasks with complete depletion of W’ at exhaustion [32].

The process through which athletes spontaneously select their pacing strategy is called teleoanticipation [20]. Teleoanticipation can be characterized as the internal “negotiation” an athlete conducts with themselves, based on the presence of a pre-determined and well-practiced pacing template, their current level of fatigue, and the anticipated distance or time remaining [19]. This internal negotiation is an almost entirely subconscious “risk analysis” that allows for PO regulation throughout a competition [1,2,3].

While objective physiological measures can be used to measure homeostatic disturbance, exercise intensity can also be appreciated through the rating of perceived exertion (RPE) [33]. RPE has been used in various settings as a subjective measure of exercise intensity at any given moment throughout an exercise bout. A higher RPE usually reflects a higher level of homeostatic disturbance (either from intensity or progressive fatigue related to the duration of an event) [2,21,22,30,31,34,35], while a lower RPE reflects a relative maintenance of homeostasis. When RPE is compared to distance of an event there is a scalar, linear growth pattern despite the occurrence of various modifiers (muscle glycogen depletion, distance, hypoxia-hyperoxia, temperature, mode of exercise) [2,8,9,19,20,21,29,30,31,34,35,36,37,38]. The association between RPE versus modulation of PO demonstrates a reciprocal relationship between transiently above-normal PO and RPE [36,37,38], supported by studies where the length of a TT was deceptively changed [37,38]. Following working at an intensity greater than normal (such as during a break away effort during a race), there is usually a reciprocal decrease in PO in order to counteract dramatic changes in homeostatic disturbance [36,38]. Similarly, if the momentary RPE is lower than expected for that point during a competition, it is likely that PO will increase.

This reciprocal relationship between RPE and changes in PO, and the abrupt decrease in PO after the starting segment of track cycling races [36,38] led to the concept of the Hazard Score (HS) which describes the likelihood that athletes will change their PO during competition, with the twin goals of avoiding catastrophic collapse during an athletic competition while optimizing performance [39,40]. The HS combines the momentary RPE and the percent distance of the race remaining as a predictor of change in PO (e.g., velocity). When an individual begins a race too quickly, they will reduce the speed in order to sustain a rate of growth of RPE [30] that will allow to finish the race without “collapsing”. The HS can also be used to calculate a potentially more powerful predictor, the summated HS, throughout an event in order to better understand the effect of accumulated fatigue on pacing during competition. Accordingly, the intent of this study was to evaluate how the summated HS grows during a simulated competitive event in relation to the starting strategy. The hypothesis was that PO would be regulated after an enforced starting strategy in a way designed to control the final value of the summated HS toward a common final value.

## 2. Materials and Methods

The subjects for this study were seven well-trained (7–10 h per week), recreational level, cyclists, age 25–61 years. The subjects were mostly long distance “tourists”, and performed limited competitive cycling, but regularly participated in “tours” of up to 160 km. Within the classification scheme of De Pauw et al. [41] and Delcroix et al. [42], they were in categories 2–3. The Physical Activity Readiness Questionnaire was completed by each subject to identify contraindications (e.g., exclusion criteria) to exercise testing. Written informed consent was provided by each subject prior to testing and the protocol was approved by the Institutional Review Board for the Protection of Human Subjects at the University of Wisconsin-La Crosse (Protocol 20.SB.080).

For subject characterization, each subject performed maximal incremental exercise on an electronically braked cycle ergometer (Lode, Groningen, Netherlands). Tests were conducted to provide peak PO, maximal oxygen uptake, ventilatory threshold, maximal heart rate, and maximal RPE. After a warm-up stage of 3-min at 25 W, PO was increased of 25 W/min until pedaling cadence could not be maintained within the range of 70–90 rpm.

Following the maximal test each subject performed a total of five 10-km (km) cycling TTs on a Velotron cycle ergometer (Velotron Electronic Bicycle Ergometer, Elite Model, Racer Mate, Seattle, WA, USA). Prior to all TTs, there was a self-selected warm-up of 15–30 min, which included 2–3 bursts of 30–60 s at the anticipated starting velocity. The first two TTs were practice 10-km TTs to allow the athletes to become habituated to the 10-km cycling TT [6]. The subsequent, randomly ordered, three TTs, were conducted in a manner in which the initial PO (3-km) was manipulated, based on the average PO of the first 3-km of the 2nd practice TT (PO_init_). During the self-paced TT, the subject was only instructed to finish the TT as quickly as possible. During the fast-start TT, the PO during the initial 3-km was 5% greater than PO_init_. During the slow-start TT, the PO during the initial 3-km was 5% less than PO_init_. This was reinforced by a visual display visible to the rider and verbal feedback from the investigator. The remaining 7-km were finished as rapidly as possible. A small monetary reward ($10), based on improving final TT performance versus the 2nd practice TT, was offered to provide a “competitive incentive” during TTs 3–5. PO was measured continuously by the ergometer, and integrated every 0.5-km. The RPE was measured every 1-km using the Category Ratio (0–10) RPE scale [33]. Blood lactate was measured every 2-km in fingertip blood using dry chemistry (Lactate Pro, Arkray, Japan). Heart rate (HR) was measured using radio telemetry with data averaging every 5 s (T31, Polar Electro Oy, Kempele, Finland). Session RPE (sRPE) was measured ~30 min after the cool-down [43].

Descriptive characteristics of subjects were calculated as mean ± standard deviation. Time and average PO of the three experimental TTs (self-paced, fast-start and slow-start) were compared using a one-way Analysis of Variance (ANOVA) with repeated measures. The HS was calculated by multiplying momentary RPE by the remaining fraction of the race [39]. Summated HS was calculated by adding the HS values from each km. Two-way ANOVA with repeated measures was used to analyze differences in lactate, RPE, PO, and HR between the three experimental TTs. Pairwise comparisons were made using Tukey’s post-hoc tests. Significance was set at *p* < 0.05 to achieve statistical significance.

## 3. Results

Descriptive data from the maximal tests are presented in Table 1. Table 2 shows differences in time, average PO, and sRPE between the three TTs. There were no statistically significant differences in finish time, average PO, and sRPE between the three experimental TTs (*p* > 0.05). On average, the self-paced TT was 15.6 s faster than the slow-start TT and 12.6 s faster than the fast-start TT. sRPE, which included the warm-up, the TT, and cool-down, was the greatest for the fast-start TT and least for the slow-start TT.

**Figure 1:**
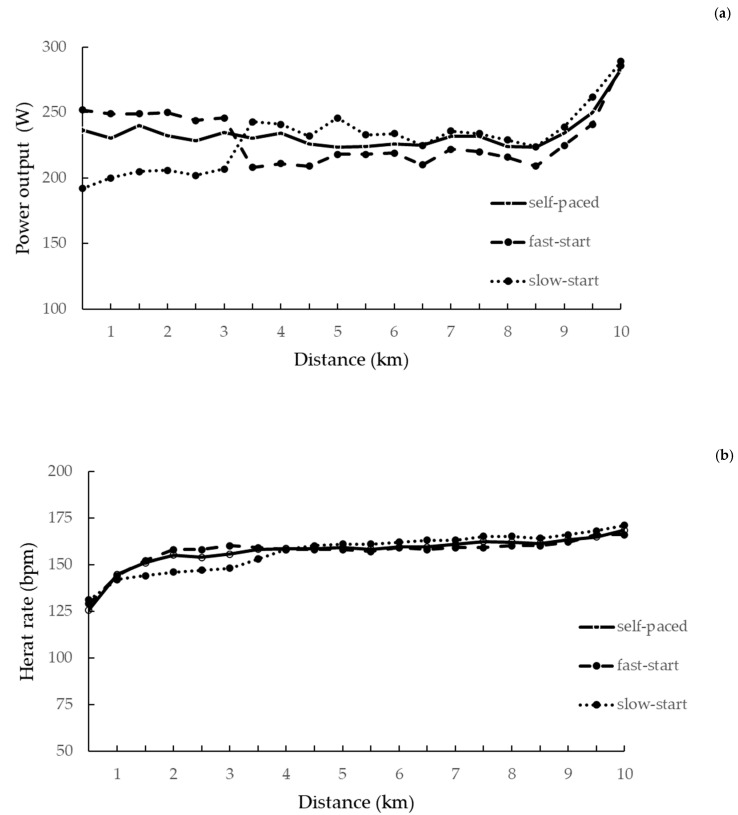

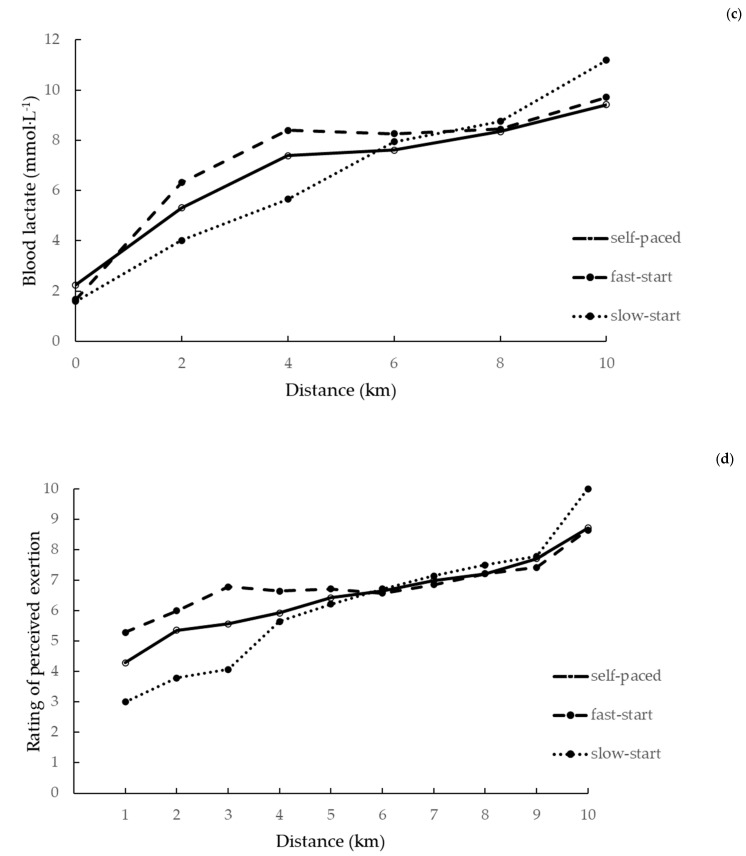
Power output (**a**), heart rate (**b**), blood lactate (**c**), and rating of perceived exertion (**d**) responses in relation to starting strategy.

The pattern of PO, RPE, blood lactate concentration, and HR within the three TTs is shown in Figure 1.

A significant interaction between the starting strategy and the distance covered was shown for PO (*p* = 0.034), RPE (*p* = 0.027), blood lactate concentration (*p* = 0.043), and HR (*p* = 0.046).

As per design of the study, the PO for the fast-start TT was significantly greater than the slow-start TT for the first 3-km. PO for the self-paced TT was significantly greater than the slow start trial at the 500-m mark (Table 3). RPE for the fast-start TT was significantly greater than the slow-start TT at kilometers 1, 2, and 3 (Table 4.). Blood lactate for the fast-start TT was significantly greater than the slow-start TT at the 4 km time point (Table 5). HR for the fast-start TT was significantly greater than the slow-start TT at kilometers 2, 2.5, and 3 (Table 6).

**Figure 2:**
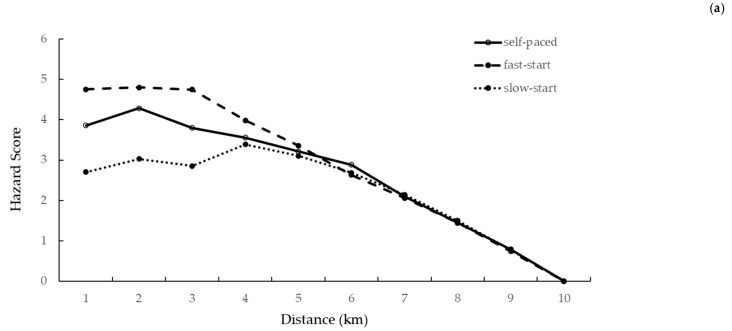

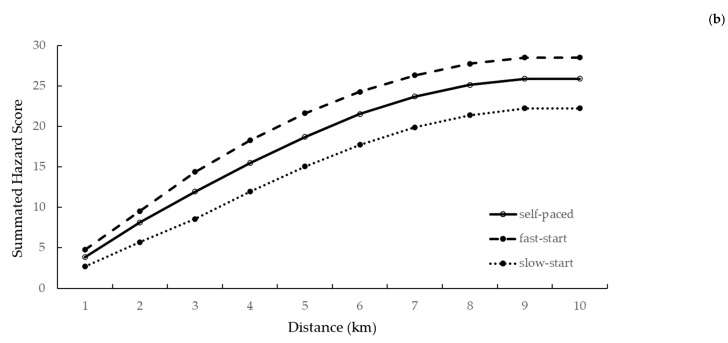
Growth of Hazard Score (**a**) and summated Hazard Score (**b**) in relation to distance during self-paced, fast-start, and slow-start time trials.

The pattern of changes in the HS and summated HS within the three TTs is shown in Figure 2.

There was a significant interaction between the starting strategy and the distance covered for HS (*p* = 0.022) and summated HS (*p* = 0.031). HS during the fast-start TT was significantly greater than the self-paced TT at kilometers 1 and 3. HS during the fast-start TT was significantly greater than the slow-start TT at kilometers 1 and 2. HS during the self-paced TT was significantly greater than the slow-start TT at kilometers 1, 2, and 3 (Table 7). Summated HS during the fast-start TT was significantly greater than the self-paced TT from kilometers 3–10. Summated HS during the fast-start TT was significantly greater than the slow-start trial for kilometers 1–10. Summated HS during the self-paced TT was significantly greater than the slow-start trial for kilometers 2–10 (Table 8).

**Figure 3:**
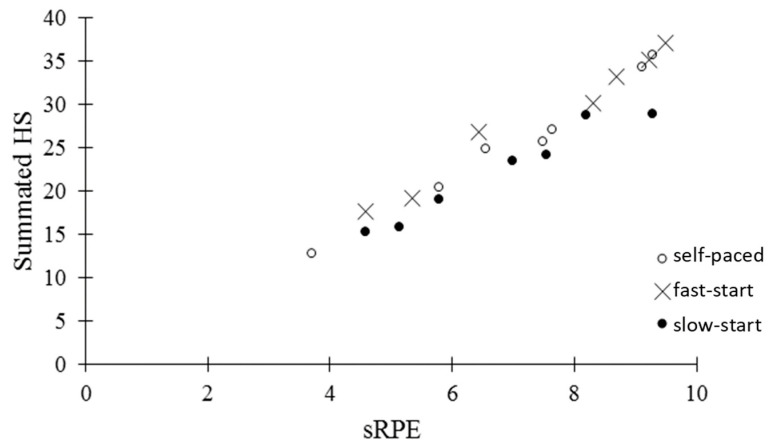
Session rating of perceived exertion (sRPE) to summated Hazard Score (HS) relationship during self-paced, fast-start, and slow-start time trials.

The relationship between the sRPE and the summated HS is presented in Figure 3. There was a strong correlation (r = 0.88), suggesting that the perceived net effort of a TT was dependent on the pattern of effort within the TT. In particular, the fast-start TT, which was slower for overall performance than the self-selected TT, produced a higher summated HS and a higher sRPE.

## 4. Discussion

The main purpose of this study was to determine whether manipulating starting strategy would affect TT performance, the summated HS, or whether the subject would change their PO so that a common value for summated HS was achieved during a simulated competition. Contrary to the hypothesis, it was found that although there was a reduction in PO following the fast-start, the summated HS remained higher compared to the self-start and slow-start strategy. This occurred despite a meaningfully slower performance time in both the fast-start and slow-start TT. This coincides with Robinson et al. [24] who concluded that it is vital to follow a relatively even pace (e.g., self-selected starting strategy) in order to avoid large homeostatic disturbances early during an event. This was supported in earlier studies performed in our laboratory [2,7,9,28,29,30,36,39]. The results are consistent with the evolution of pacing strategy to a more even pattern during contemporary 1-mile world records [28], and in events where individuals bettered their own best performance [9].

The importance of these data is reflected in the 2008 Olympic pace data of Thiel et al. [7] who showed that some runners in Olympic finals would run with the leaders for part of the race before suddenly dropping off the leading pace and finding a relatively constant individual pace which allowed them to finish the race, usually with an end-spurt. This mirrors the PO pattern observed in the fast-start TT in the current data and the large reduction in PO after a “break away” effort [28]. In light of the present findings, these data can be interpreted as suggesting that once a critical summated HS is achieved, the PO will require reduction, but that the reduction in PO will not be adequate to force the summated HS toward a common terminal value.

In the present study, the behavior of the summated HS was reflected by the strong correlation between the summated HS and sRPE (r = 0.88). This corresponds with Cohen et al. [36] who showed if RPE is above that usually observed at a specific point during an event, such as after a break away effort, PO will decrease in order to accommodate to, and recover from, large changes in homeostasis. When the RPE comes back into the usually observed scalar pattern of growth, PO returns to the normal profile. Similar results were observed by Schallig et al. [38] in trials where subjects were deceived regarding the duration of the trial. Immediately after being told that a trial was going to be longer than anticipated, subjects reduced PO until the rate of increase of RPE returned to what it normally would have been in the longer trail. Although previous research has shown that athletes tend to follow a predetermined template where the rate of increase of RPE is adjusted to the distance of the race remaining in order to avoid disturbances in homeostasis [30,31], it may be that PO is the variable that is manipulated while RPE continues to increase in a linear fashion. This was also shown in the report of Joseph et al. [30] and Henslin-Harris et al. [44] where the blinded administration of an inhaled hypoxic gas mixture lead to a reduction in PO without changing the rate of growth of RPE. Other studies have shown that there is a linear relationship between RPE and relative duration of exercise despite exercise conditions [2]. Similar results were shown by Baldassare et al. [40] who found that despite differences in pacing strategies (positive pacing versus even pacing), RPE increased or only slightly decreased similarly with each strategy. Athletes apparently change their pace to match RPE to an anticipated growth pattern, so although HS may be the same between trials at a specific time point, the summated HS would be higher with a faster start.

In the present study, while summated HS was different between TTs, the finish time was not significantly different (although 15 s is a time difference of large practical magnitude) reflecting that since knowledge of the endpoint was present and distance remaining was not relatively large, the athletes were able to generate an end spurt despite the large summated HS. RPE has been shown to increase when athletes are aware of the distance to the endpoint [30,31]. This is also reflected by Foster et al. [6] who showed a significant difference between finishing a race quickly and the ability to maintain a constant, high PO for an extended period of time.

Optimal race performance is not always about who has the highest PO, but who can maintain optimal PO in order to perform a successful end spurt. de Koning et al. [45] compared the effect of various pacing strategies on performance in 1000 and 4000 m track cycling, showing that even small changes in pacing strategy led to changes in performance. This highlighted the importance of pacing strategy in the pursuit of competitive success. While the best time for the 1000 m TT was obtained by the cyclist with the highest peak PO (all-out strategy), the fastest time for the 4000 m was attained with a faster start followed by a constant PO after ~12 s (even pace). Time can also be augmented by the athlete’s interaction with the environment. Konings et al. [8] utilized virtual opponents starting either +3% or −1% compared to a familiarization trial. Results showed that even in a lab setting, the use of virtual opponents led to faster performances, showing that the self-selected pacing trial has to be slightly faster than previously attempted in order for the athletes to improve performance. This can be applied to athletes who begin a race too fast leading to an accumulation of fatigue posing potentially, detrimental effects to their performance. More generally, pacing in a way that does not use an unrealistically high PO early within an event can aid in successful athletic competition. It is evident that many high-level athletes may begin a race quickly in order to match the pace of their competitors [7]. This information will also assist in determining the ideal PO for starting in order to optimize performance. However, it must be recognized that to improve performance, an athlete must take a “calculated risk”, which often involves a faster start than normal. In many cases, they may develop too much discomfort (e.g., high summated HS) and fail to improve their time. In other cases, this may lead to small improvements in performance which are athletically important.

In this particular study, the specific application was to a maximal effort TT. However, an equivalent argument may be made toward training bouts. Very high early PO can lead to increases in RPE [46] and lead to reduced enjoyment during the training bout [23], which carries the risk that adherence to an exercise program is likely reduced.

One limitation to this study included a limited sample size and task habituated tourist type cyclists rather than experienced TT athletes. Although we have shown that task habituation leads to stable performances [6], more accomplished athletes might deliver somewhat different, and more specifically relevant, results. Future studies should also evaluate the difference of the summated HS in shorter and longer distance events to see how the relationship between RPE and PO differ from a middle-distance event. Further, the effect of PO sequencing within normal training bouts should be considered, relative to adherence to the exercise prescription.

## 5. Conclusions

The results of this study indicate that despite a reduction in PO following a fast-start TT, the summated HS remains higher with a fast-start strategy. This indicates that summated HS is a powerful predictor to better understand accumulated fatigue on pacing pattern during simulated competition. The sum of all HS from the beginning of the race to the present point have a cumulative effect on the outcome of the event, the physiological state, and the sRPE experienced by the exerciser.

## Figures and Tables

**Table 1:** Means ± standard deviations of the descriptive characteristics of men and women during maximal incremental exercise testing.

Characteristic	Male (n = 5)	Female (n = 2)
Age (years)	39.0 ± 3.71	45.0 ± 11.31
Height (cm)	176.8 ± 3.90	166.4 ± 1.80
Weight (kg)	81.6 ± 11.48	62.0 ± 5.66
VO_2max_ (L/min)	4.1 ± 0.39	3.1 ± 0.82
Peak PO (W)	305 ± 44.7	237 ± 17.7
PO at VT (W)	165 ± 22.4	138 ± 17.7
HR_max_ (bpm)	170 ± 7.4	163 ± 0.0

VO_2max_: maximal oxygen uptake; PO: power output; VT: ventilatory threshold; HR_max_: maximal heart rate.

**Table 2:** Means ± standard deviations of time, average power output (PO), and session rating of perceived exertion (sRPE) of self-paced, fast-start, and slow-start trials.

Variable	Self-Paced	Fast-Start	Slow-Start
Time (min)	17.51 ± 1.41	17.72 ± 1.87	17.77 ± 1.74
Average PO (W)	234 ± 62.6	230 ± 62.0	230 ± 62.7
sRPE	7.1 ± 1.94	7.4 ± 1.97	6.8 ± 1.70

**Table 3:** Means ± standard deviations of power output (W) during self-paced, fast-start, and slow-start time trials.

Distance (km)	Self-Paced	Fast-Start	Slow-Start
0.5	237 ± 76.4 *	252 ± 50.6 *	192 ± 68.7
1.0	231 ± 60.0	249 ± 57.2 *	200 ± 59.5
1.5	240 ± 54.8	249 ± 57.2 *	205 ± 53.1
2.0	232 ± 62.0	250 ± 56.5 *	206 ± 53.4
2.5	229 ± 56.5	244 ± 52.1 *	202 ± 54.0
3.0	235 ± 68.1	246 ± 51.7 *	207 ± 53.0
3.5	230 ± 68.4	208 ± 79.8	243 ± 61.0
4.0	234 ± 68.1	211 ± 82.4	241 ± 60.0
4.5	226 ± 52.5	209 ± 78.2	232 ± 57.2
5.0	224 ± 62.6	218 ± 76.6	246 ± 66.7
5.5	224 ± 68.6	218 ± 78.4	233 ± 73.8
6.0	226 ± 71.9	219 ± 76.1	234 ± 70.4
6.5	225 ± 61.7	210 ± 63.0	225 ± 63.5
7.0	232 ± 68.8	222 ± 72.6	236 ± 70.1
7.5	232 ± 72.4	220 ± 73.9	234 ± 72.9
8.0	224 ± 66.5	216 ± 69.6	229 ± 61.1
8.5	223 ± 61.5	209 ± 64.6	224 ± 68.1
9.0	235 ± 65.6	225 ± 72.2	239 ± 81.7
9.5	250 ± 62.4	241 ± 73.3	262 ± 71.7
10.0	283 ± 83.0	286 ± 96.8	289 ± 87.4

* Significantly greater than slow-start trial.

**Table 4:** Means ± standard deviations of rating of perceived exertion (RPE) during self-paced, fast-start, and slow-start time trials.

Distance (km)	Self-Paced	Fast-Start	Slow-Start
1	4.3 ± 1.38	5.3 ± 1.38 *	3.0 ± 0.82
2	5.4 ± 1.75	6.0 ± 1.83 *	3.8 ± 1.15
3	5.6 ± 1.90	6.8 ± 1.63 *	4.1 ± 1.30
4	5.9 ± 2.21	6.6 ± 1.97	5.6 ± 1.49
5	6.4 ± 1.99	6.7 ± 2.06	6.2 ± 1.58
6	6.6 ± 2.17	6.6 ± 2.44	6.7 ± 1.80
7	7.0 ± 2.08	6.9 ± 2.12	7.1 ± 1.49
8	7.2 ± 1.91	7.2 ± 2.16	7.5 ± 1.55
9	7.7 ± 1.89	7.4 ± 2.30	7.8 ± 1.82
10	8.7 ± 1.50	8.6 ± 1.55	10.0 ± 4.42

* Significantly greater than slow-start trial.

**Table 5:** Means ± standard deviations of blood lactate concentration (mmol∙L^−1^) during self-paced, fast-start, and slow-start time trials.

Distance (km)	Self-Paced	Fast-Start	Slow-Start
0	2.2 ± 1.24	1.7 ± 0.46	1.6 ± 0.54
2	5.3 ± 2.60	6.3 ± 3.22	4.0 ± 1.12
4	7.4 ± 2.60	8.4 ± 2.88 *	5.7 ± 1.26
6	7.6 ± 3.25	8.3 ± 2.64	7.9 ± 2.02
8	8.3 ± 3.37	8.4 ± 2.70	8.8 ± 2.21
10	9.4 ± 2.53	9.7 ± 2.61	11.2 ± 2.06

* Significantly greater than slow-start trial.

**Table 6:** Means ± standard deviations of heart rate (bpm) during self-paced, fast-start, and slow-start time trials.

Distance (km)	Self-Paced	Fast-Start	Slow-Start
0.5	126 ± 19.2	129 ± 23.0	131 ± 26.0
1.0	145 ± 11.8	144 ± 17.0	142 ± 15.8
1.5	151 ± 9.0	152 ± 8.5	144 ± 10.1
2.0	155 ± 8.0	158 ± 6.7 *	146 ± 8.5
2.5	154 ± 6.9	158 ± 7.9 *	147 ± 9.5
3.0	156 ± 5.4	160 ± 8.0 *	148 ± 5.8
3.5	158 ± 6.5	159 ± 4.9	153 ± 5.1
4.0	159 ± 6.5	158 ± 3.8	158 ± 5.4
4.5	158 ± 7.1	158 ± 6.1	160 ± 4.0
5.0	159 ± 7.0	158 ± 5.3	161 ± 4.6
5.5	158 ± 6.6	157 ± 5.7	161 ± 4.9
6.0	159 ± 6.9	159 ± 6.2	162 ± 4.9
6.5	159 ± 6.7	158 ± 5.3	163 ± 4.2
7.0	161 ± 7.5	159 ± 4.0	163 ± 4.7
7.5	162 ± 6.4	159 ± 4.4	165 ± 3.6
8.0	162 ± 6.2	160 ± 4.9	165 ± 3.8
8.5	161 ± 7.1	160 ± 3.9	164 ± 4.8
9.0	163 ± 5.5	162 ± 4.5	166 ± 6.0
9.5	165 ± 5.2	166 ± 6.4	168 ± 5.9
10.0	168 ± 5.4	166 ± 6.4	171 ± 6.7

* Significantly greater than slow-start trial.

**Table 7:** Means ± standard deviations of the Hazard Score during self-paced, fast-start, and slow-start time trials.

Distance (km)	Self-Paced	Fast-Start	Slow-Start
1	3.9 ± 1.24 *^#^	4.8 ± 1.24	2.7 ± 0.73 *
2	4.3 ± 1.40 ^#^	4.8 ± 1.46	3.0 ± 0.92 *
3	3.8 ± 1.45 *^#^	4.8 ± 1.14	2.9 ± 0.91
4	3.6 ± 1.32	3.9 ± 1.18	3.4 ± 0.90
5	3.2 ± 0.99	3.4 ± 1.03	3.1 ± 0.79
6	2.9 ± 0.88	2.6 ± 0.98	2.7 ± 0.72
7	2.1 ± 0.62	2.1 ± 0.63	2.1 ± 0.45
8	1.4 ± 0.38	1.4 ± 0.43	1.5 ± 0.31
9	0.8 ± 0.16	0.7 ± 0.23	0.8 ± 0.18
10	0.0 ± 0.00	0.0 ± 0.00	0.0 ± 0.00

* Significantly less than fast-start trial; ^#^ Significantly greater than slow-start trial.

**Table 8:** Means ± standard deviations of the summated Hazard Score during self-paced, fast-start, and slow-start time trials.

Distance (km)	Self-Paced	Fast-Start	Slow-Start
1	3.9 ± 1.24	4.8 ± 1.24 *	2.7 ± 0.73
2	8.1 ± 2.59 *	9.6 ± 2.65 *^#^	5.7 ± 1.61
3	11.9 ± 3.96 *	14.4 ± 3.76 *^#^	8.6 ± 2.50
4	15.5 ± 5.23 *	18.3 ± 4.73 *^#^	12.0 ± 3.27
5	18.7 ± 6.16 *	21.7 ± 5.64 *^#^	15.1 ± 4.02
6	21.6 ± 6.85 *	24.3 ± 6.49 *^#^	17.8 ± 4.69
7	23.7 ± 7.33 *	26.3 ± 7.08 *^#^	19.9 ± 5.11
8	25.1 ± 7.68 *	27.8 ± 7.45 *^#^	21.4 ± 5.40
9	25.9 ± 7.85 *	28.5 ± 7.66 *^#^	22.3 ± 5.65
10	25.9 ± 7.85 *	28.5 ± 7.66 *^#^	22.3 ± 5.65

* Significantly greater than slow-start trial; ^#^ Significantly greater than self-paced trial.

## Data Availability

The data presented in this study are available on request from the corresponding author.
